# Response of wheat aphid to insecticides is influenced by the interaction between temperature amplitudes and insecticide characteristics

**DOI:** 10.3389/fphys.2023.1188917

**Published:** 2023-04-24

**Authors:** Kun Xing, Shu-Ming Zhang, Mei-Qi Jia, Fei Zhao

**Affiliations:** ^1^ Shanxi Key Laboratory of Integrated Pest Management in Agriculture, College of Plant Protection, Shanxi Agricultural University, Taiyuan, China; ^2^ Shanxi Shouyang Dryland Agroecosystem National Observation and Research Station, Shouyang, China

**Keywords:** global warming, insecticides, multiple stress, risk assessment, temperature amplitude, temperature characteristics

## Abstract

**Introduction:** Climate change not only directly affects the phenotype of organisms but also indirectly impacts their physiology, for example, by altering their susceptibility to insecticides. Changed diurnal temperature fluctuations are an important aspect of climate change; ignoring the impact of these fluctuations on the biological effects of various chemical insecticides can lead to inaccurate assessments of insecticide risk under the current and future climate change scenarios.

**Methods:** In this study, we studied effects of different temperature amplitudes (± 0, ± 6, ± 12°C) at the same mean temperature (22°C) on the life history traits of a globally distributed pest (*Sitobion avenae*, wheat aphid), in response to low doses of two insecticides. The first, imidacloprid shows a positive temperature coefficient; the second, beta-cypermethrin has a negative temperature coefficient.

**Results:** Compared with the results seen with the constant temperature (22°C), a wide temperature amplitude (± 12°C) amplified the negative effects of imidacloprid on the survival, longevity, and fecundity of *S. avenae*, but significantly increased the early fecundity of the wheat aphid. Beta-cypermethrin positively impacted the wheat aphid at all temperature amplitudes studied. Specifically, beta-cypermethrin significantly increased the survival, longevity, and fecundity of *S. avenae* under medium temperature amplitude (± 6°C). There were no significant differences in the survival, longevity, and the early fecundity of *S. avenae* when it was treated with beta-cypermethrin at the wide temperature amplitude (± 12°C). However, the negative effect of beta-cypermethrin on the intrinsic rate of increase of *S. avenae* decreased gradually with the increase in temperature amplitude.

**Discussion:** In conclusion, the response of *S. avenae* to positive temperature coefficient insecticides was markedly affected by temperature amplitude, while negative temperature coefficient insecticides increased the environmental adaptability of *S. avenae* to various temperature amplitudes. Our results highlight the importance of the integrated consideration of diurnal temperature fluctuations and different temperature coefficient insecticide interactions in climate-change-linked insecticide risk assessment; these results emphasize the need for a more fine-scale approach within the context of climate change and poison sensitivity.

## 1 Introduction

Climate change (Körner and Basler, 2010) and the application of insecticides (Rohr et al., 2011), as two major anthropogenic pressures, have brought developmental and reproductive challenges to a wide array of organisms (Landis et al., 2014). These factors have increased research interest to improve our understanding of the ecophysiological mechanisms that underly the biological responses of organisms to climate change and insecticides (Van den Brink et al., 2018). Currently, there are three different viewpoints with regards to this issue. The first viewpoint suggests that factors related to climate change can increase biological sensitivity to insecticide pollutants, which is referred to as, climate-induced toxicity sensitivity (CITS) (Noyes et al., 2009; Noyes and Lema, 2015). The second viewpoint proposes that exposure to insecticide pollutants can increase biological sensitivity to climate change, known as toxication-induced climate change sensitivity (TICS) (Moe et al., 2013; Janssens et al., 2018). The third viewpoint asserts that these concepts do not exist independently owing to inherent differences between species (De Beeck et al., 2018). Overall, more experimental evidence is necessary to fully understand the biological interactions between climate change and insecticide pollution.

Studies have established that temperature remarkably affects the insecticide mortality of insects. For example, a rise in temperature has been shown to increase insect sensitivity to organophosphorus, nicotine, and benzoylurea (Lydy et al., 1999; Amarasekare and Edelson, 2004). By contrast, a rise in temperature was shown to decrease insect sensitivity to pyrethroids (Harwood et al., 2009; Ma et al., 2011). Moreover, the interaction between temperature and insecticides can significantly affects the life-history traits of insects. For example, these interactions have been shown to increase the mortality of subsequent life stages (Janssens et al., 2018), decrease anti-predation behavior (Tran et al., 2019), and decrease longevity and fecundity (Zuo et al., 2015). However, some studies also found that the interaction between temperature and insecticides may increase adult reproduction (Cheng et al., 2014), alleviate oxidative damage (Janssens et al., 2018), and improve the viral diffusion of disease-carrying insects (Muturi and Alto, 2011). The delayed effect of this interaction effect can even be transmitted across generations, impacting the survival (Tran et al., 2018), development, reproduction (Zuo et al., 2015), and the anti-predation ability of offspring (Tran et al., 2019). Notably, most thermostatic studies ignore the negative effects of daily high temperature, resulting in an overestimation of the ecological adaptation of insects to ambient temperature (Arambourou and Stoks, 2015; Decourten and Brander, 2017); however, studies that focus on short-term heat shock, do not reflect the natural 24-h diurnal temperature fluctuations (Gu et al., 2010; Colinet et al., 2015; Stoks et al., 2017). Furthermore, these studies are not in line with gradual temperature changes that usually characterize most insect habitats (Mitchell and Hoffmann, 2010), and neglects the repair (within the context of thermal damage) that some insects can experience under suitable low temperatures at night (Zhao et al., 2014). Some studies have focused on the effects of temperature fluctuations and insecticide exposure on insects. Compared with a constant temperature, the interaction of temperature fluctuations and insecticides in the context of insect development, growth, insecticide sensitivity, and stage specificity (Delnat et al., 2019a; Verheyen and Stoks, 2019a; Delnat et al., 2019b; Verheyen and Stoks, 2019b) has been shown to have additional (and significant) effects on the ecophysiology of insects. However, these studies mostly focused on insecticides with positive temperature coefficient (such as chlorpyrifos and bioinsecticides), ignored the effect of insecticides with negative temperature coefficient. Furthermore, the number of comparative studies investigating the interaction between temperature fluctuations and the temperature coefficients of insecticides is currently limited. Therefore, these findings may not be sufficient to comprehensively explain the complex effects of temperature fluctuations and insecticide exposure on insects.

*Sitobion avenae* (Fabricius), one of the most important agricultural pests, is widely distributed globally (Dean, 1970; Winderet al., 1999). Owing to its small size, fast heat conduction, and high metabolic rate, *Sitobion avenae* is extremely sensitive to environmental temperature changes, making it an ideal model organism for studying the impact of environmental changes. With regards to environmental changes, diurnal temperature fluctuations have been shown to significantly impact the development, survival (Xing et al., 2021), longevity, reproduction (Zhao et al., 2014), temperature tolerance (Zhao et al., 2019), and population dynamics (Zhu et al., 2019) of various organisms. Studies examining the interaction between temperature and insecticides have primarily focused on the lethal effects of insecticides under constant temperatures (Pei et al., 2015). However, the potential impacts of interactions between temperature fluctuations and insecticides on insect populations remain poorly understood. The neonicotinoid insecticide imidacloprid (positive temperature coefficient) and the pyrethroid insecticide beta-cypermethrin (negative temperature coefficient) are commonly used in the field control of aphids (Yu et al., 2012; Su et al., 2015). Specifically, these insecticides have been widely used in the wheat-producing regions of China for a long time (Wu et al., 2011; Dong et al., 2020). A better understanding of the combined effects of diurnal temperature fluctuations and insecticides are critical not only for guiding the safe and effective use of insecticides in the context of global warming but also for accurately assessing their potential ecological impacts.

Therefore, herein, we studied the effect of the interaction between temperature amplitudes and different temperature coefficient insecticides (solvent control, imidacloprid, and beta-cypermethrin) on the life history traits of *S. avenae* (i.e., survival, longevity, fecundity, early fecundity and the intrinsic rate of increase).

## 2 Materials and methods

### 2.1 Insects

*Sitobion avenae* was collected in May 2016 from within the wheat experimental field of Shanxi Academy of Agricultural Sciences (116°16′N, 35°55′E). They were then reared on 10–20 cm winter wheat, which was replaced once a week by winter wheat seedlings. The pots with winter wheat were placed inside a breathable and transparent insect-rearing cage (60 cm × 60 cm × 60 cm). The cages were finally placed in the insect rearing room, under the following conditions: 22°C ± 1°C, 50%–60% relative humidity, and 16-h light/8-h dark photoperiod, lights on from 05:00 to 21:00 and lights off from 21:00 to 05:00. *Sitobion avenae* was reared indoors for at least 3 years before the experiment commenced.

### 2.2 Temperature design

In this study, temperature data from the National Meteorological Information Centre (http://data.cma.cn/en) for May (between 2012 and 2018), which corresponded to the rapid growth period of wheat, in Linfen, northern China were selected. Preliminary analysis of the data revealed that the average daily temperature during the rapid growth period of wheat was approximately 22°C, with a mean diurnal temperature amplitude of ± 6°C and a maximum diurnal temperature amplitude of ± 12°C (Supplementary Figure S1) (Xing et al., 2021). Therefore, in this study, we used 22°C as the average temperature, while the diurnal temperature variation was set to ± 0, ± 6, and ± 12°C, respectively. The actual temperature and humidity in the artificial climate chambers (RXZ-380B-LED; Ningbo Jiangnan Instrument Factory, Ningbo, China) were automatically recorded at 20-min intervals in each group of variable temperature settings using a Datalogger (Hobo u23-001; Onset Computer Corporation, Bourne, MA, United States) During the experiment, target temperature average and amplitudes are 22 ± 0, 22 ± 6, and 22°C ± 12°C, recorded average temperatures (mean ± SD) are 22.39°C ± 0.13°C, 21.21°C ± 0.26°C and 21.85°C ± 0.20°C, recorded temperature amplitudes (mean ± SD) are 0.89°C ± 0.20°C, 6.89°C ± 0.15°C and 11.93°C ± 0.28°C (Supplementary Table S1, Supplementary Figure S2) (Xing et al., 2021). The photoperiod and relative humidity of the artificial climate chambers, in each group of variable temperature settings, was set as 16-h light/8-h dark, and 50%–60%, respectively. The laboratory temperature was controlled by central air conditioning and kept at a constant temperature of approximately 22°C.

### 2.3 Insecticide concentration

In this study, a positive temperature coefficient insecticide (PT, imidacloprid), a negative temperature coefficient insecticide (NT, beta-cypermethrin), and a solvent control (SC, distilled water) were selected for wheat aphid control in wheat fields. Imidacloprid and beta-cypermethrin, with a purity grade of >99% for both insecticides, were supplied by Yangzhou Suling Insecticide Chemical Co. Ltd. (Yangzhou, China). We selected the dose at which 10% of the individuals will die (LC_10_) for these two insecticides for their application. Imidacloprid (6 mg) and cypermethrin (0.5 mg) were dissolved in Tween 80 (0.5 mL) and acetone (4.5 mL), both analytically pure, to obtain 1.2 mg/mL imidacloprid and 0.1 mg/mL cypermethrin stock solutions, respectively, and then diluted with distilled water to obtain their serial dilutions (imidacloprid: 1.2, 3.6, 10.8, 32.4, and 97.2 μg/mL; cypermethrin: 0.1, 0.5, 2.5, 12.5, and 62.5 μg/mL). Using SC as the reference, the toxicity of these insecticides to 9-day-old aphids were measured following a Food and Agriculture Organization of the United Nations (FAO) recommended spotting method, and the number of surviving aphids was recorded after 24 h. Each treatment was replicated three times with 30 insects per replicate. The data were analyzed using probit analysis in the SPSS 21.0 software (SPSS Inc., Chicago, IL, United States). The regression equation of toxicity was obtained as imidacloprid: y = −0.817 + 1.011x, *χ* ^*2*^ = 1.401, *df* = 5, n = 720; beta-cypermethrin: y = −0.121 + 0.755x, *χ* ^*2*^ = 1.128, *df* = 5, n = 720. The sublethal concentrations of imidacloprid and beta-cypermethrin were determined as LC_10_ = 0.347 μg/mL (95% confidence interval (CI) 0.055–0.895) and LC_10_ = 0.029 μg/mL (95% CI 0.003–0.096), respectively.

### 2.4 Test procedure

**FIGURE 1:**
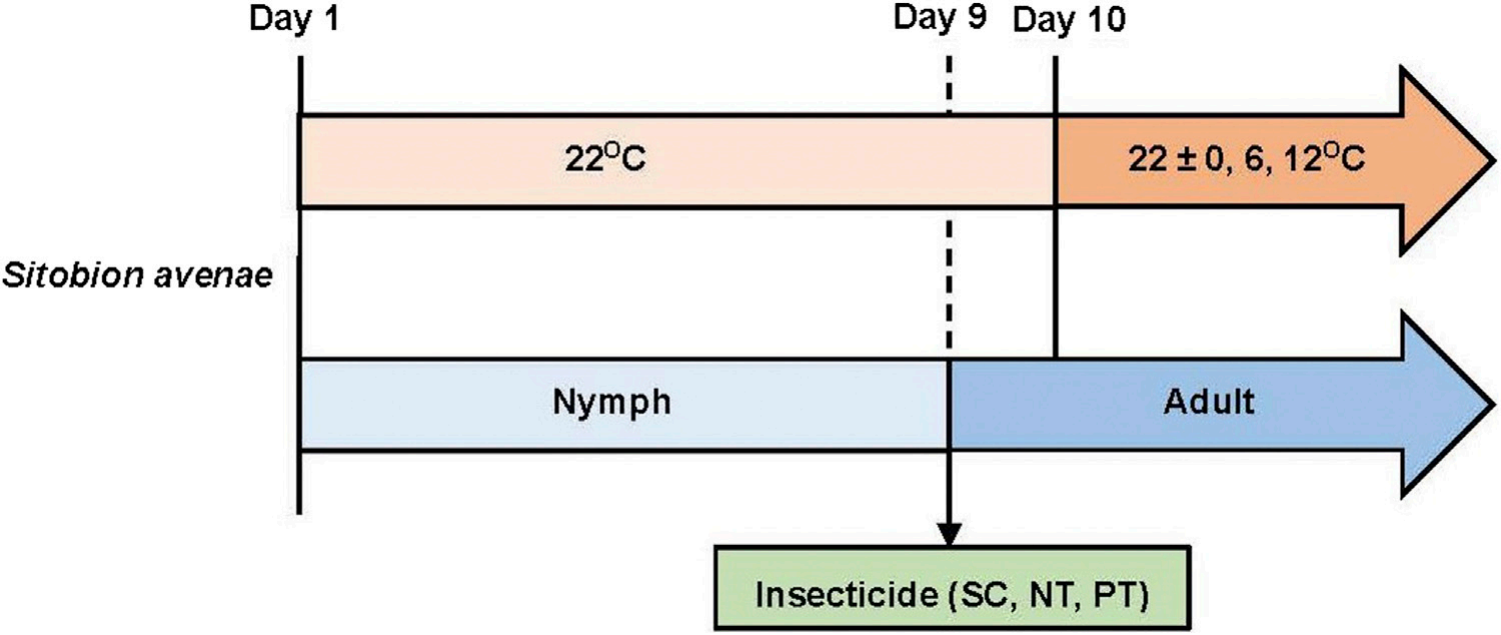
The experimental design including treatments and endpoints used.

A total of 432 nymphs that were born within 4 h were selected and reared individually in a rearing unit consisting of a plastic tube (diameter 15 mm, length 70 mm) sealed using a sponge plug, with a 10 mm slit holding a newly excised wheat leaf (Cao et al., 2021). and maintained at 22°C. On day 9, aphids (most of which had developed into adults) were subjected to three insecticide treatments (i.e., SC, PT, and NT) at three temperature amplitude (22 ± 0, 22 ± 6, and 22°C ± 12°C), with 48 adults per treatment (Figure 1). Individual mortality and reproduction were assessed daily at 08:00, and dead aphids and newly born nymphs were removed until all test aphids were dead, and the experiment was completed. To ensure adequate nutrient supply, wheat leaves in the feeding tubes were replaced every 2 days.

### 2.5 Statistical analyses

In this study, the longevity is determined as the time from day 10 until death of an adult after treatment; the fecundity is the total number of offspring produced by an adult after day 10 until death; the early fecundity is the proportion of the number of offspring produced in the first 3 days to the total number of offspring. All data were tested for normality using the Shapiro-Wilk test. We found that most of the data were skewed. Therefore, we used the gamma error distribution in generalized linear models for a two-factor analysis of the adult longevity, fecundity and early fecundity (i.e., insecticide, temperature amplitude, and their interactions) and the Fisher’s least significant difference procedure for multiple comparisons between treatments. The overall survival of adults was analyzed using a Cox proportional hazard model with two factors (insecticide and temperature amplitude) and their interactions. The survival curves of the adult between treatments under a single factor were analyzed using the Log-rank approach with the Kaplan-Meier analysis, and multiple comparisons between the two were made using the Holm-Sidak method. All data were analyzed using SPSS 21.0 (SPSS Inc., Chicago, IL, United States).

The intrinsic rate of increase (r_m_) was calculated as follows: R_0_ = ∑lx*mx, G = ∑lx*mx*X/∑lx*mx, r_m_ = ln (R0)/G, where “*X*” is the age of the adult, lx is the proportional survival of the adult at age “*X*”, and “mx” is the number of offspring per the adult at age “*X*”. We used the bootstrap procedure in R to analyze the mean and 95% CI of the intrinsic rate of increase of *S. avenae* population. Kruskal–Wallis multiple comparisons were also performed between treatments using the “Kruskal” function in the “agricolae” data package of R (Zhu et al., 2019).

## 3 Results

### 3.1 Effects of interaction between temperature amplitudes and insecticides on survival

**FIGURE 2:**
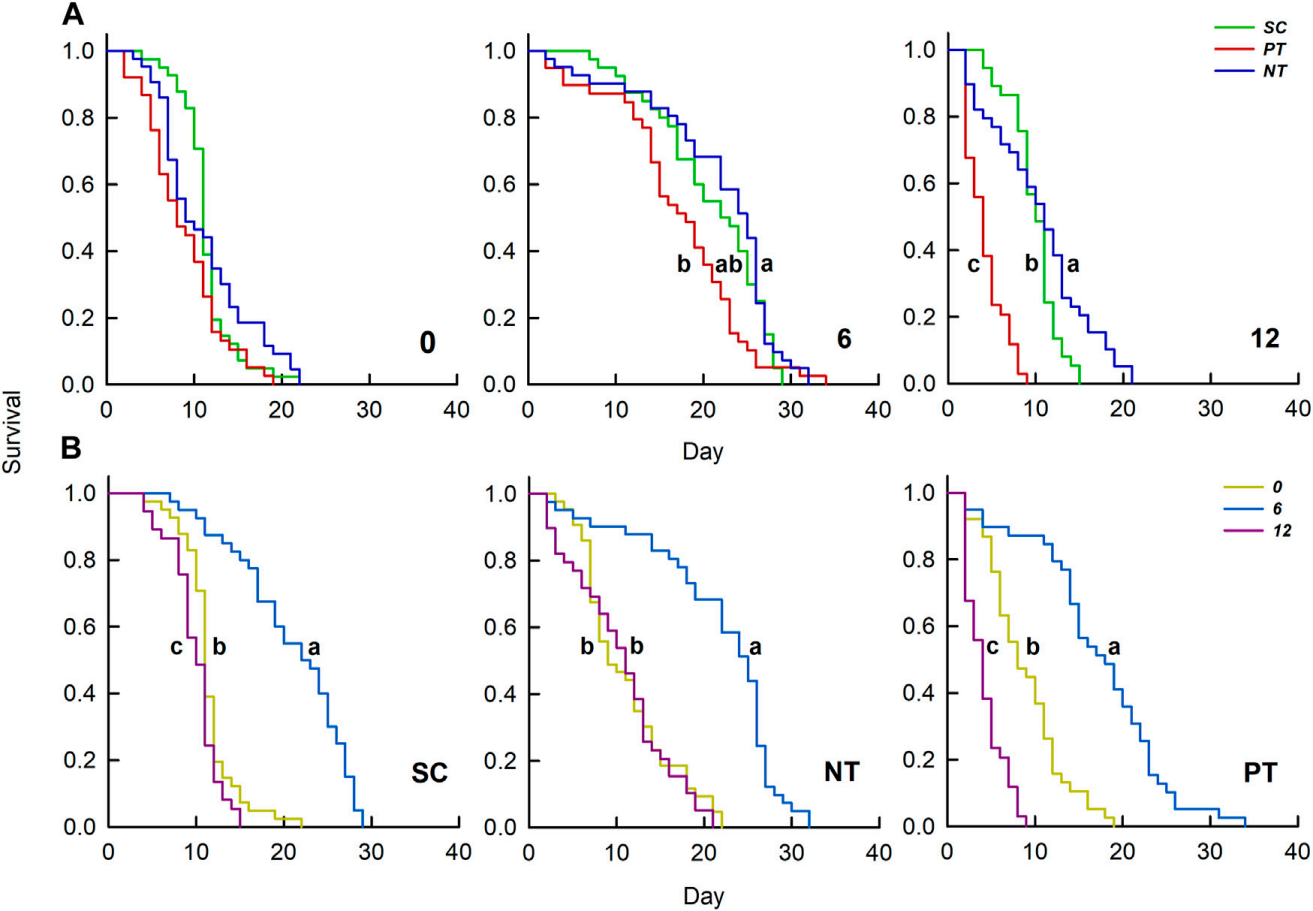
Effects of insecticides **(A)** and temperature amplitudes **(B)** on the survival curve. Different letters above the curve indicate significant differences between treatments (*p* = 0.05); 0, 6, and 12 represent the different temperature amplitudes; SC, NT, and PT represent the control, high chlorine, and imidacloprid treatment, respectively.

Temperature amplitudes (*χ* ^*2*^ = 73.73, *df* = 2, *p* < 0.001) had a significant effect on survival, whereas insecticides (*χ* ^*2*^ = 5.64, *df* = 2, *p* = 0.060) had no significant effect on survival. However, the interaction of these two factors had a significant effect on survival (*χ* ^*2*^ = 25.78, *df* = 4, *p* < 0.001) (Table 1; Figure 2). Under temperature amplitude ± 0°C, insecticides had no significant effect on survival (*χ* ^*2*^ = 6.54, *df* = 2, *p* = 0.083). However, insecticides significantly affected survival at the temperature amplitude ± 6 and ± 12°C (*χ* ^*2*^ = 9.36, *df* = 2, *p* = 0.009; *χ* ^*2*^ = 69.49, *df* = 2, *p* < 0.001), and the survival with the NT treatment was significantly higher than that with the PT treatment. Under SC, NT, and PT treatments, temperature amplitudes had a significant effect on survival (*χ* ^*2*^ = 75.43, *df* = 42, *p* < 0.001; *χ* ^*2*^ = 62.90, *df* = 42, *p* < 0.001; *χ* ^*2*^ = 93.24, *df* = 42, *p* < 0.001, respectively), and survival at the temperature amplitude ± 6°C was significantly higher than that at ± 0 and ± 12°C.

**TABLE 1:** Results of the temperature amplitude and insecticide effects on *Sitobion avenae*.

Trait	Source	*χ2*	*Df*	*p*
Survival	Temperature amplitudes (TA)	73.73	2	<0.001
	Insecticide treatments (IT)	5.64	2	0.060
	TA x IT	25.78	4	<0.001
Longevity	Temperature amplitudes (TA)	333.00	2	<0.001
	Insecticide treatments (IT)	47.68	2	<0.001
	TA x IT	6.27	4	0.180
Fecundity	Temperature amplitudes (TA)	190.12	2	<0.001
	Insecticide treatments (IT)	39.34	2	<0.001
	TA x IT	7.18	4	0.127
Early fecundity	Temperature amplitudes (TA)	189.20	2	<0.001
	Insecticide treatments (IT)	37.65	2	<0.001
	TA x IT	18.63	4	<0.001
Intrinsic rate of increase	Temperature amplitudes (TA)	12820.61	2	<0.001
	Insecticide treatments (IT)	861.48	2	<0.001
	TA x IT	1967.31	4	<0.001

### 3.2 Effects of interaction between temperature amplitudes and insecticides on longevity

**FIGURE 3:**
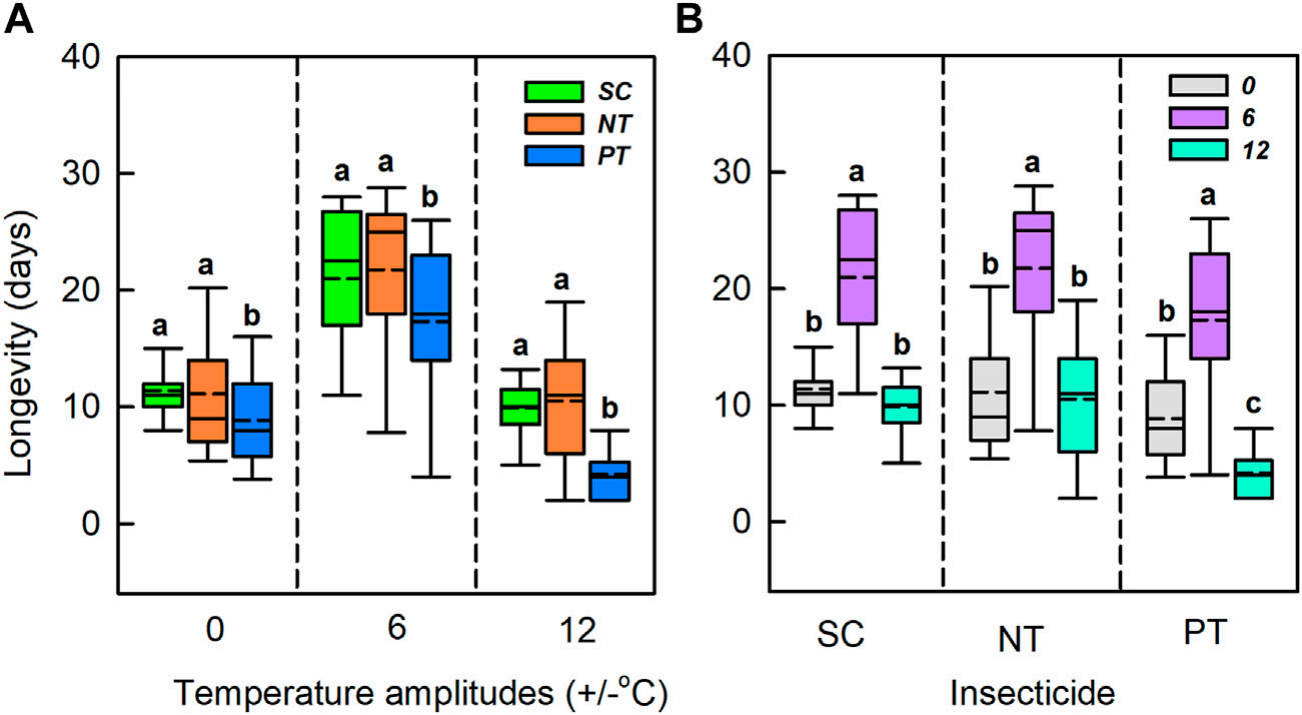
Box plot of the longevity under various temperature amplitudes **(A)** and insecticides **(B)**. The upper and lower boundaries of the box indicate the 75th percentile and 25th percentile of the dataset. The black horizontal line and short dash line within the box represent the median and mean values, respectively. Error bars above and below the box indicate the minimum and maximum values, respectively. Different letters above each box indicate significant differences (*p* < 0.05) among the treatments.

Temperature amplitudes (*χ* ^*2*^ = 333.00, *df* = 2, *p* < 0.001) and insecticides (*χ* ^*2*^ = 47.68, *df* = 2, *p* < 0.001) had significant effects on longevity, whereas their interaction had no significant effect on longevity (*χ* ^*2*^ = 6.27, *df* = 4, *p* = 0.180) (Table 1; Figure 3). Under the temperature amplitude ± 0°C, insecticides had a significant effect on longevity (*χ* ^*2*^ = 8.79, *df* = 2, *p* = 0.012). Longevity treated with NT and PT was lower than that treated with SC (0.3 and 2.6 days, respectively). At the temperature amplitude ± 6 and ± 12°C, insecticides had significant effects on longevity (*χ* ^*2*^ = 11.37, *df* = 2, *p* = 0.003; *χ* ^*2*^ = 41.13, *df* = 2, *p* < 0.001). Longevity with NT treatment was higher than that with SC treatment (increased by 0.9 and 0.6 days, respectively), and longevity with PT treatment was lower than that with SC treatment (decreased by 3.7 and 5.7 days, respectively). With the SC, NT, and PT treatments, the temperature amplitude had significant effect on longevity (*χ* ^*2*^ = 54.13, *df* = 2, *p* < 0.001; *χ* ^*2*^ = 44.59, *df* = 2, *p* < 0.001; *χ* ^*2*^ = 56.81, *df* = 2, *p* < 0.001, respectively). Moreover, longevity with the SC, NT and PT treatments, under the temperature amplitude ± 6°C was higher than those at the temperature amplitude ± 0°C (9.6, 10.6, 8.5 days, respectively), and longevity at the temperature amplitude ± 12°C was lower than that at temperature amplitude ± 0°C (1.5, 0.6, 4.6 days, respectively).

### 3.3 Effects of interaction between temperature amplitudes and insecticides on fecundity

**FIGURE 4:**
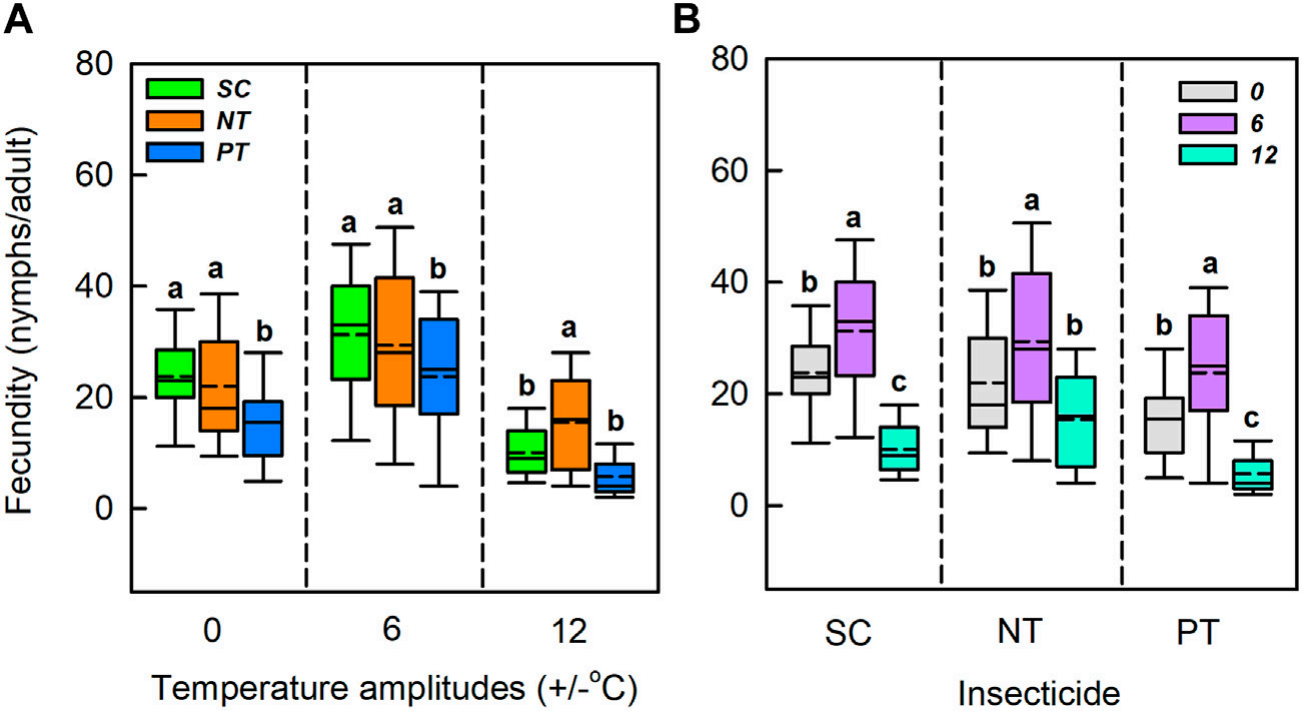
Box plot of the fecundity under various temperature amplitudes **(A)** and insecticides **(B)**. The upper and lower boundaries of the box indicate the 75th percentile and 25th percentile of the dataset. The black horizontal line and short dashed-line within the box represent the median and mean values, respectively. Error bars above and below the box indicate the minimum and maximum values, respectively. Different letters above each box indicate significant differences (*p* < 0.05) among the treatments.

Temperature amplitudes (*χ* ^*2*^ = 190.12, *df* = 2, *p* < 0.001) and insecticides (*χ* ^*2*^ = 39.34, *df* = 2, *p* < 0.001) had significant effects on fecundity, whereas their interaction had no significant effect on fecundity (*χ* ^*2*^ = 7.18, *df* = 4, *p* = 0.127) (Table 1; Figure 4). Under the temperature amplitude ± 0°C, insecticides significantly affected fecundity (*χ* ^*2*^ = 17.35, *df* = 2, *p* < 0.001). Fecundity with NT and PT treatment was lower than that with SC treatment (1.8 nymphs/adult and 8.3 nymphs/adult, respectively). At the temperature amplitude ± 6°C, insecticides significantly affected fecundity (*χ* ^*2*^ = 7.31, *df* = 2, *p* = 0.026), and fecundity treated with NT and PT was lower than that treated with SC (1.86 nymphs/adult and 7.5 nymphs/adult, respectively). At the temperature amplitude ± 12°C, insecticides significantly affected fecundity (*χ* ^*2*^ = 29.69, *df* = 2, *p* < 0.001). Fecundity observed under NT treatment was higher than that under SC treatment (increased by 5.4 nymphs/adult), and fecundity observed under PT treatment was lower than that under SC treatment (decreased by 4.4 nymphs/adult). Under the SC, NT, and PT treatments, temperature amplitudes had significant effect on fecundity (*χ* ^*2*^ = 61.93, *df* = 2, *p* < 0.001; *χ* ^*2*^ = 21.28, *df* = 2, *p* < 0.001; *χ* ^*2*^ = 51.02, *df* = 2, *p* < 0.001; respectively). The fecundity under the temperature amplitude ± 6°C, with the SC, NT, and PT treatments, was higher than that under the temperature amplitude ± 0°C (7.5 nymphs/adult, 7.4 nymphs/adult and 8.3 nymphs/adult, respectively), and fecundity treated with temperature amplitude ± 12°C was lower than that of temperature amplitude ± 0°C (13.7 nymphs/adult, 6.6 nymphs/adult and 9.9 nymphs/adult, respectively).

### 3.4 Effects of interaction between temperature amplitudes and insecticides on early fecundity

**FIGURE 5:**
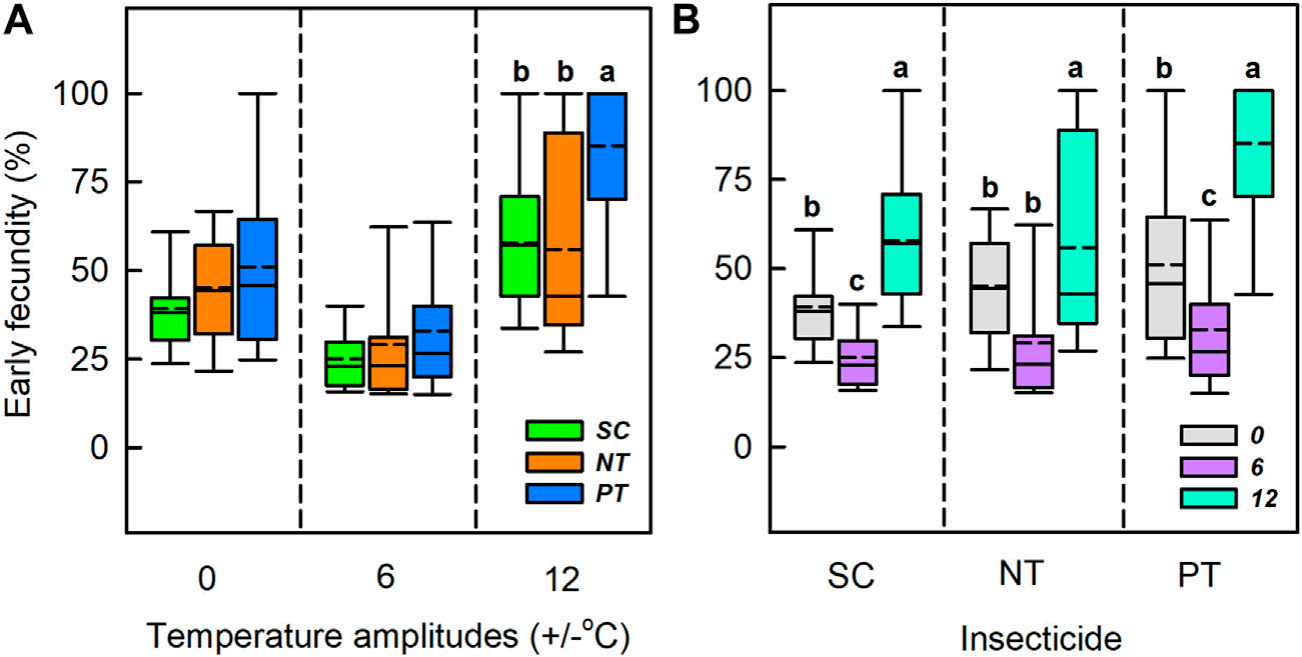
Box plot of early fecundity under various temperature amplitudes **(A)** and insecticides **(B)**. The upper and lower boundaries of the box indicate the 75th percentile and 25th percentile of the dataset. The black horizontal line and short dashed-line within the box represent the median and mean values, respectively. Error bars above and below the box indicate the minimum and maximum values, respectively. Different letters above each box indicate significant differences (*p* < 0.05) among the treatments.

Temperature amplitudes (*χ* ^*2*^ = 189.21, *df* = 2, *p* < 0.001), insecticides (*χ* ^*2*^ = 37.65, *df* = 2, *p* < 0.001), and their interaction (*χ* ^*2*^ = 18.63, *df* = 4, *p* < 0.001) had a significant effect on the early fecundity (Table 1; Figure 5). Under the temperature amplitudes ± 0 and ± 6°C, insecticides had no significant effect on the early fecundity (*χ* ^*2*^ = 5.65, *df* = 2, *p* = 0.059; *χ* ^*2*^ = 2.99, *df* = 2, *p* = 0.225). At ± 12°C, insecticides significantly affected the early fecundity (*χ* ^*2*^ = 23.30, *df* = 2, *p* < 0.001), and the effect of NT treatment was lower than that of SC treatment (1.93% lower), whereas the early fecundity with PT treatment was higher than with SC treatment (27.26% higher). Under the SC, NT, and PT treatments, temperature amplitudes had significant effect on the early fecundity (*χ* ^*2*^ = 57.52, *df* = 2, *p* < 0.001; *χ* ^*2*^ = 36.29, *df* = 2, *p* < 0.001; *χ* ^*2*^ = 51.55, *df* = 2, *p* < 0.001). The early fecundity at the temperature amplitude ± 6°C, with the SC, NT, and PT treatments, was lower than that at the temperature amplitude ± 0°C (decreased by 14.2%, 16.0% and 18.2%, respectively), while the early fecundity at temperature amplitude ± 12°C was higher than that at temperature amplitude ± 0°C (increased by 18.6%, 10.9%, and 34.1%, respectively).

### 3.5 Effects of interaction between temperature amplitudes and insecticides on the intrinsic rate of increase

**FIGURE 6:**
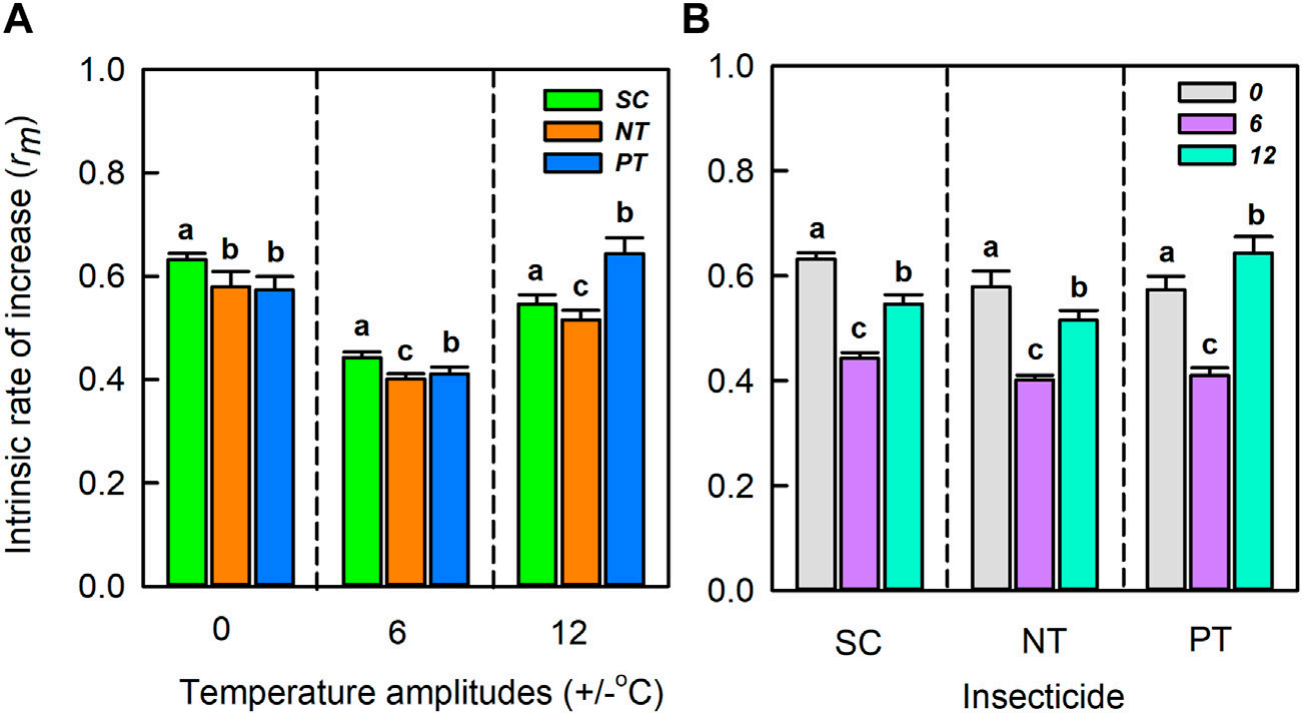
The Intrinsic rate of increase (mean ± SD) under temperature amplitudes **(A)** and insecticides **(B)**. Different lowercase letters represent significant differences between different treatments at the *p* = 0.05 level.

Temperature amplitudes (*χ* ^*2*^ = 12820.61, *df* = 2, *p* < 0.001) and insecticides (*χ* ^*2*^ = 861.48, *df* = 2, *p* < 0.001), including their interaction (*χ* ^*2*^ = 1967.31, *df* = 2, *p* < 0.001), significantly affected the intrinsic rate of increase (Table 1; Figure 6). At the temperature amplitude ± 0 and ± 6°C, the intrinsic rate of increase was significantly affected by insecticides (*χ* ^*2*^ = 161.702, *df* = 2, *p* < 0.001; *χ* ^*2*^ = 198.620, *df* = 2, *p* < 0.001). The intrinsic rate of increase with the NT treatment was low than that with SC treatment (0.05 and 0.04, respectively), and the intrinsic rate of increase with PT treatment was lower than that with SC treatment (0.06 and 0.03, respectively). At the temperature amplitude ± 12°C, the intrinsic rate of increase was significantly affected by insecticides (*χ* ^*2*^ = 234.90, *df* = 2, *p* < 0.001). The intrinsic rate of increase was higher with PT treatment than with SC treatment (increased by 0.10) and lower with PT treatment than with control SC treatment (decreased by 0.03). Under SC, NT, and PT treatments, the intrinsic rate of increase was significantly affected by temperature amplitudes (*χ* ^*2*^ = 263.11, *df* = 2, *p* < 0.001; *χ* ^*2*^ = 253.21, *df* = 2, *p* < 0.001; *χ* ^*2*^ = 256.80, *df* = 2, *p* < 0.001, respectively). The intrinsic rate of increase at the temperature amplitude ± 6°C with SC, NT, PT treatments was lower than that at the temperature amplitude ± 0°C (decreased by 0.19, 0.18, and 0.16, respectively). The intrinsic rate of increase at the temperature amplitude ± 12°C with SC and NT was lower than that at the temperature amplitude ± 0°C (decreased by 0.09, 0.06 respectively), but the intrinsic rate of increase at the temperature amplitude ± 12°C with PT was higher than that at the temperature amplitude ± 0°C (increased by 0.07).

## 4 Discussion

### 4.1 Effects of insecticides alone

Under constant temperature, insecticides had a delayed effect on adult longevity, fecundity, and the intrinsic rate of increase. Overall, these results are consistent with previous studies about the effects of low-dose insecticides on life history traits of insects (Lu et al., 2016; Imelda Martiner et al., 2017). We also found that the delay in the negative effects of imidacloprid on adult longevity and fecundity was much more profound than that of beta-cypermethrin at the same low dose. This may be due to the different correlations between specific insecticides and temperature fluctuations (Nadia et al., 2018). Previous studies have shown that imidacloprid has a positive temperature coefficient and virulence increases with increasing temperature (Gonias et al., 2008; Liu et al., 2016). The toxicity of beta-cypermethrin decreased gradually with an increase in temperature, showing a negative correlation (Musser and Shelton., 2005; Harwood et al., 2009). In this study, the constant temperature of 22°C may have increased adults’ susceptibility to imidacloprid, and it may be closely related to immune gene expression, detoxification enzyme and acetylcholine lipase activity (Fan et al., 2021).

### 4.2 Effects of temperature amplitudes alone

The temperature amplitude significantly affected the phenotype and population parameter of the adult. Compared with constant temperature and medium temperature amplitude (22°C ± 6°C), the wide temperature amplitude 22°C ± 12°C (maximum daily temperature up to 34°C) reduced adult longevity and fecundity, but the adults still survived long enough to reproduce. This result is discordant with the result of previous studies on the correlation between insect survival and constant temperature. *Sitobion avenae* could not survive at 30°C (Dean, 1974; Jiang et al., 2018). In our experiments, the adult longevity was reduced to 9.9 days and the fecundity was reduced to 10.1 nymphs/adult at the wide temperature amplitude 22°C ± 12°C. These trends show that an adult could still survive and have relatively highly fecundity even when the maximum daily temperature reached 34°C. This may be owing to the repair effect of protective substances such as heat shock proteins, mannitol, and sorbitol accumulated during the suitable recovery temperature after the adults experienced a daily high temperature (Yang and Stamp, 1995). The early fecundity with wide temperature amplitude 22°C ± 12°C was significantly higher than that under the constant temperature (22°C ± 0°C) and medium temperature amplitude (22°C ± 6°C), which may be caused by the response of the adult to high temperature and pressure. High daily temperatures (34°C) with the wide temperature amplitude (22°C ± 12°C) as a temperature-pressure may cause the adult to accelerate reproduction of offspring to maximize fitness, this change in reproductive strategies is adaptive (Javoiš and Tammaru, 2004).

Compared with the constant temperature (22°C ± 0°C) and wide temperature amplitude (22°C ± 12°C), medium temperature amplitude (22°C ± 6°C) increased adult survival, extended the adult longevity, and promoted fecundity. Studies have shown that appropriate temperature amplitudes can accelerate development, improve survival and temperature tolerance, increase fecundity and ability to transmit poison and olfactory response, and so on (Colinet et al., 2015). Our study showed that suitable temperature amplitude significantly increased the survival, extended the longevity, and increased the fecundity. This finding may be explained by the fact that the appropriate temperature amplitude is closely related to the increase in the activity or concentration of key proteins (heat shock proteins), protective agents, and so on, in the metabolic processes of the insects (Colineta et al., 2007; Lalouettea et al., 2011).

### 4.3 Effects of the interactions between temperature amplitudes and insecticides

The negative effect of imidacloprid on the adult was further intensified under temperature fluctuations. Compared with the results seen with constant temperature, the wide temperature amplitude of 22°C ± 12°C significantly inhibited the survival, longevity, and fecundity of the adult; moreover, medium temperature amplitude (22°C ± 6°C) significantly reduced the increases in the adult phenotypic parameters. This shows the toxicity of insecticides increases as temperature amplitudes increased. Studies have shown that the exposure of organisms to insecticides can increase their biological sensitivity to climate change; this phenomenon is known as TICS (Janssens et al., 2018; Verheyen et al., 2019). This may be due to the conversion of insecticides to more toxic metabolites at wide temperature amplitudes (Harwood et al., 2009), reducing the adult longevity and fecundity. It is also possible that under a high daily temperature, the higher metabolic rate of the adult leads to increased respiration and eating to compensate for energy expenditure. This high uptake of poisons, especially by those with a high metabolic conversion rate, will offset the increased rates of detoxification and excretion at high temperatures, which may precipitate increased toxicity (Hooper et al., 2013; Hallman and Brooks, 2015). The intrinsic rate of increase includes the contributions of initial reproductive age, reproductive peak, reproductive duration, and population survival. Population dynamics are one of the main factors that predict the growth potential of insect populations. Compared to our results seen with constant temperature, the interaction between temperature amplitudes and imidacloprid markedly affected the intrinsic rate of increase. Under the wide temperature amplitude, imidacloprid was associated with a high intrinsic rate of increase, due to the short adult longevity and increased early fecundity (Ahn and Choi., 2022). In conclusion, both the individual fitness of the adult and the response of population growth to positive temperature coefficient insecticides were considerably affected by the temperature amplitude.

Unexpectedly, we found that beta-cypermethrin profoundly affected adults across the different temperature amplitudes. Specifically, we found that beta-cypermethrin significantly improved survival, longevity, and fecundity of the adult at the medium temperature amplitude (22°C ± 6°C). At the wide temperature amplitude (22°C ± 12°C), the adult treated with beta-cypermethrin was not significantly affected compared to those with SC treatment, and the negative effect of beta-cypermethrin on the intrinsic rate of increase decreased gradually with the increase in temperature amplitudes. This finding disagrees with the results of previous studies, which have shown that wide temperature amplitudes can increase the toxicity of insecticides (Hooper et al., 2013; Van et al., 2014). For example, under high temperature fluctuations, chlorpyrifos increased the mortality of the mosquito (*Culex pipiens*) larvae (Delnat et al., 2019a), and it also increased the mortality of damselfly (*Ischnura elegans*) (Verheyen et al., 2019). However, our results showed that the toxicity of beta-cypermethrin to the adult was very weak at temperature amplitudes (22°C ± 6°C and 22°C ± 12°C). First, there may be a close relationship with the temperature properties of insecticides. Previous studies have shown that the effectiveness of beta-cypermethrin is negatively correlated with increases in the temperature (Harwood et al., 2009). In this study, the daily high temperature increased (up to 34°C) at the wide temperature amplitude (22°C ± 12°C), which greatly limited the effectiveness of beta-cypermethrin. Second, when organisms respond to one stressor by increasing energy expenditure, it may have a synergistic effect on their response to another stressor (Liess et al., 2016). Insecticides and environmental stress can yield coevolution in the target insects (Delnat et al., 2019b). In this study, the daily high temperature reached 34°C at the wide temperature amplitude (22°C ± 12°C), which far exceeded the upper limit of the development threshold of wheat aphid (30°C). When the adult resisted the environmental stress pressure, the synergistic effect of temperature and insecticides might be generated to reduce the toxic effect of high chlorine levels on the adult. In conclusion, in the interaction between the temperature amplitude and the negative temperature coefficient insecticide (beta-cypermethrin), the individual or population fitness of the adult improved, indicating that the negative temperature coefficient insecticide improved the environmental adaptability of insects to the temperature fluctuations.

### 4.4 Risk assessment of insecticides under climate change

By studying the effects of temperature amplitude and insecticide exposure on insect survival, longevity, fecundity, and population growth, we demonstrated that diurnal temperature fluctuations encountered by most organisms in nature are a key environmental factor that shaped toxicity-linked responses. First, we found that the insecticide toxicity measured at constant temperature is not only inconsistent with the actual conditions in the field but also independent of the interaction of global climate change and ecotoxicology on insects. Second, we demonstrated that insecticides could alter biological sensitivity to climate change and that the temperature amplitude is an important component of TICS. Finally, our study showed that wide temperature amplitude conditions significantly increased the virulence of insecticides with positive temperature coefficients and not of those with negative temperature coefficients. This suggests that the temperature properties of insecticides are also crucial for assessing the risk of insecticide virulence under current and future climate scenarios. Overall, our results highlight the importance of combining the temperature amplitudes with the temperature characteristics of insecticides to improve the accuracy and prediction of insecticide risk assessments under global climate change (Rasmussen et al., 2018; Delnat et al., 2019b; Verheyen et al., 2019).

## Data Availability

The original contribution presented in the study are included in the article/Supplementary Material, further inquiries can be directed to the corresponding author.
