# Supplementary figures and images for: Response of wheat aphid to insecticides is influenced by the interaction between temperature amplitudes and insecticide characteristics
Source: Front Physiol. 2023 Apr 24;14:1188917. doi: 10.3389/fphys.2023.1188917 (PMC10165072; doi:10.3389/fphys.2023.1188917)

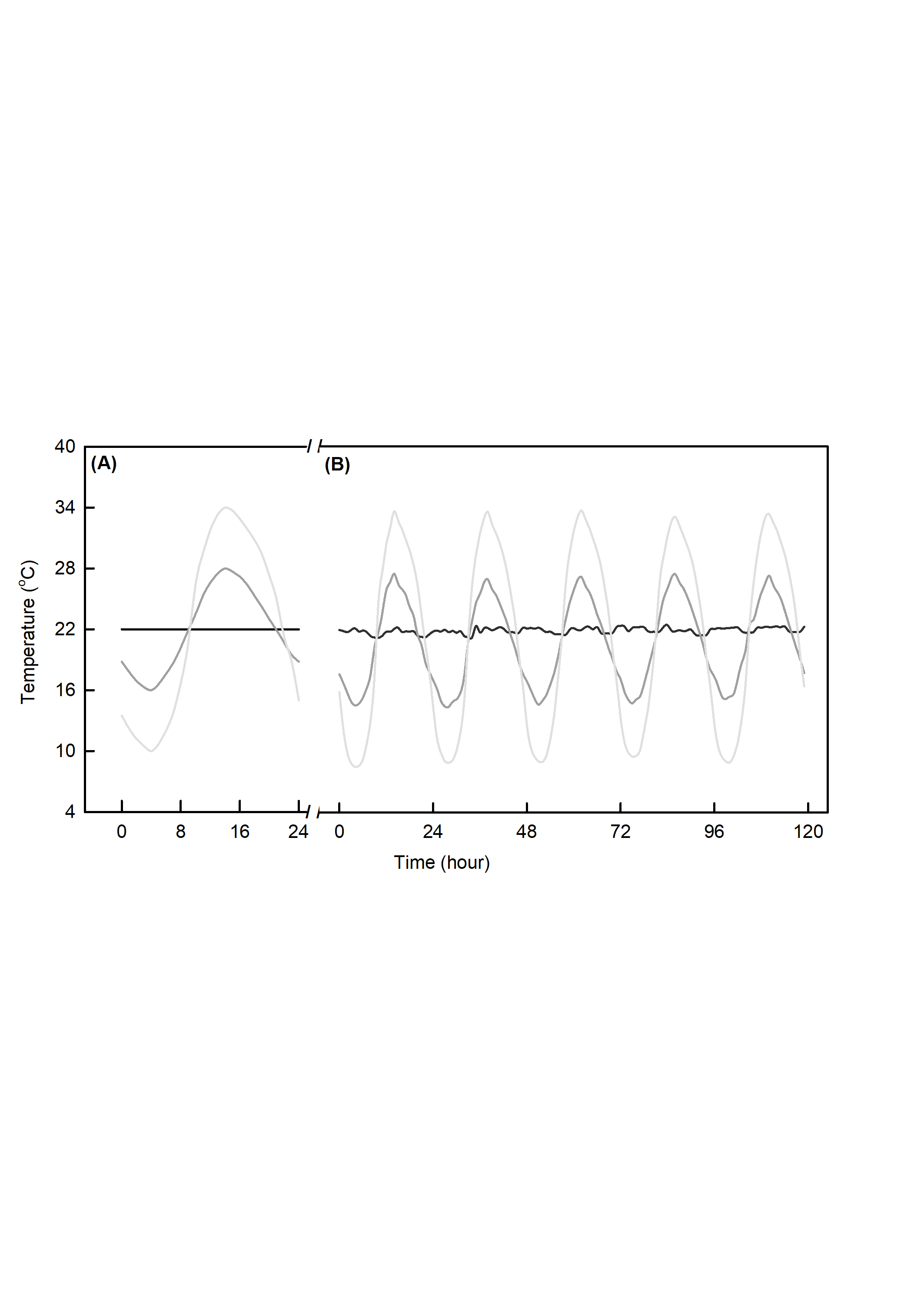

Supplement: Supplementary file 1 [file Image2.jpg]

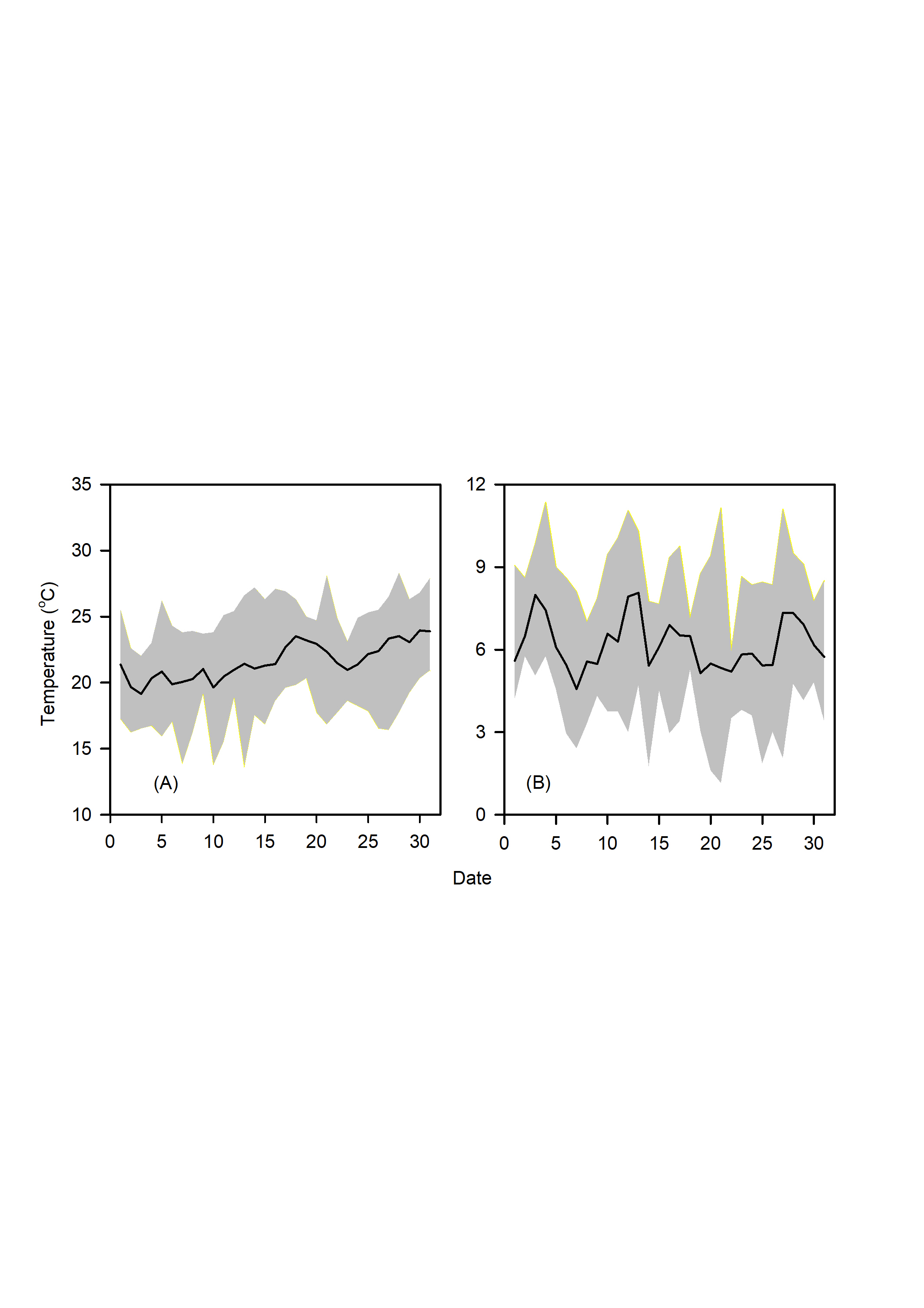

Supplement: Supplementary file 3 [file Image1.jpg]
