# Supplementary material for: Response of wheat aphid to insecticides is influenced by the interaction between temperature amplitudes and insecticide characteristics
Source: Front Physiol. 2023 Apr 24;14:1188917. doi: 10.3389/fphys.2023.1188917 (PMC10165072; doi:10.3389/fphys.2023.1188917)
Supplement: Supplementary file 2 [file Table1.doc]

**Supplement Table 1** Target and actual recorded temperatures with different temperature amplitudes around 22 °C in climate chambers

| Target temperature range (± °C) | Recorded temperature (mean ± SD) | |
| --- | --- | --- |
|  | Average (°C) | Temperature amplitudes (°C) |
| 0 | 22.39 ± 0.13 | 0.89 ± 0.20 |
| 6 | 21.21 ± 0.26 | 6.89 ± 0.15 |
| 12 | 21.85 ± 0.20 | 11.93 ± 0.28 |
